# Temperature and Humidity Regulate Sporulation of *Corynespora cassiicola* That Is Associated with Pathogenicity in Cucumber (*Cucumis sativus* L.)

**DOI:** 10.3390/biology11111675

**Published:** 2022-11-18

**Authors:** Qian Zhao, Yanxia Shi, Yikai Wang, Xuewen Xie, Lei Li, Tengfei Fan, Liyun Guo, Ali Chai, Baoju Li

**Affiliations:** 1 Institute of Vegetables and Flowers, Chinese Academy of Agricultural Sciences, Beijing 100081, China; 2 Department of Plant Pathology, China Agricultural University, Beijing 100094, China; 3 Science and Technology Research Center of China Customs, Beijing 100026, China

**Keywords:** cucumber (*Cucumis sativus*), cucumber target leaf spot, spore production, spore size, virulence

## Abstract

**Simple Summary:**

Cucumber is an important vegetable crop for mankind. Cucumber target leaf spot, caused by *Corynespora cassiicola*, is a worldwide disease. The disease is commonly treated with fungicides, but due to the emergence of resistance, the efficacy of fungicides has declined. At present, control of this disease has been problematic. Spores of *C*. *cassiicola* play a significant role in the epidemiology of the disease. Owing to this, fungus cultivation is necessary to properly understand its life cycle as well as pathogenesis for the possible future development of an efficient and environmentally friendly control method. Therefore, in this study, different temperatures and moistures were evaluated on four substrates to see their impact on the sporulation of *C*. *cassiicola*. Furthermore, the relationship between spore size and pathogenicity was determined. The results showed that temperature and moisture affected not only spore production, but also spore size. In addition, spore size was found to be an important virulence determinant in *C*. *cassiicola*.

**Abstract:**

Cucumber target leaf spot, caused by *Corynespora cassiicola*, is an emerging disease with a high incidence that causes severe damage to cucumbers on a global scale. Therefore, efforts need to be undertaken to limit the spread and infection of this pathogen, preferably by using environmentally friendly methods. In this study, the effects of temperature and moisture on the sporulation of *C*. *cassiicola* were investigated in vitro and in vivo. The novelty of our study refers to the observation of spore production and size as well as the revelation of a correlation between spore size and virulence. On potato dextrose agar (PDA) and cucumber−leaf extract agar (CEA), temperature played a critical role in spore production, which was strongly influenced by both temperature and moisture on detached leaves and cucumber seedlings. Maximum spore production was found at 30 °C on PDA and 25 °C on CEA, cucumber detached leaves and living plants. Lower spore productions were observed with a stepwise change of 5 °C. In addition, the largest spore production was found at 100% relative humidity (RH) in comparison to the other tested moisture. Moreover, moisture was found to be the most important factor affecting spore size, accounting for 83.09–84.86% of the total variance in length and 44.72–73.10% of the total variance in width. The longest−narrowest spores were formed at 100% RH, and the shortest−widest spores were formed at 75% RH. Furthermore, the result showed that larger spores of *C. cassiicola* were more virulent and small spores were avirulent. Our findings will contribute to the development of new strategies for the effective alleviation and control of cucumber target leaf spot.

## 1. Introduction

*Corynespora cassiicola* (Berk. & M. A. Curtis) C. T. Wei, as a necrotrophic phytopathogenic ascomycetous fungus, can infect more than 530 plant species from 380 genera, occurring on leaves, stems, fruits, and roots [1,2]. The fungus was first reported as *Helminthosporium cassiicola* (as ‘*cassiaecola*’) on *Cassia* in Cuba [3] and later observed on cucumber and named *Corynespora mazei* by Güssow [4]. These two names and other synonyms were combined with *Corynespora cassiicola* by Wei [5].

Cucumber target leaf spot (TLS) disease, caused by *C. cassiicola*, is one of the most destructive diseases in more than 70 countries, including the United States [6], Mexico [7], South Korea [8], and Japan [9]. Disease symptoms include initial pallid to yellowish−brown spots with a necrotic center and dark brown margins that appear at random on the leaves. As the disease progresses, lesions coalesce and produce large irregular necrotic areas ranging from less than 2 mm to 2 cm in diameter. In the past ten years, outbreaks of cucumber TLS disease occurred in Hebei, Liaoning, Shandong, Jiangsu, Shanxi, and Guangxi provinces and the Inner Mongolia Autonomous Region of China [10,11], especially when overcast and rainy days were coupled with excessive overwintering inoculum and large acreage of susceptible cultivars. The disease incidence on cucumber plants varies from 20% to 70% in different fields, causing 30–80% yield losses [12,13,14]. To prevent crop loss, many chemical fungicides have been applied during the growing season, which in turn increases the resistance of pathogens to fungicides and alters the population genetics of the pathogens [6,9].

Sporulation of fungal pathogens plays a crucial role in epidemics of plant diseases. Some plant pathogens can be spread by wind and rainfall, in which air currents and splashing droplets carry pathogenic spores within and among susceptible host plants [15,16,17]. Thus, fungal spores are responsible for initiating new disease cycles. The relationship between sporulation and epidemics is convincingly described by Nelson and Tung [18]: “No one portion of the disease cycle exerts a greater influence on epidemic increase of many diseases than the production of inoculum for subsequent infection.” Previous studies have shown that plant pathogens such as rust spores can be transported long distances (kilometers) in the atmosphere and even across continents [19,20,21]. On wet fruit, the incidence of gray mold increases from 0.1 to 83.1% as the airborne concentration of *Botrytis cinerea* spores increase from 0 to 8.6 per liter of air [22]. *C. cassiicola* is an ascomycete fungus that primarily exists as asexual spores (conidia) and vegetative mycelia in nature. The infection is caused by conidia, and penetration hyphae developed from germ tubes invade healthy plants directly or via stomata and wounds [12]. Under favorable conditions, large numbers of spores are produced on lesions, blighted tissues, and plant debris; later, secondary spread occurs by spores that are mainly transmitted by air currents after being liberated following a reduction in relative humidity (RH) [23] and occasionally with splashed water [24]. In sesame fields, a positive correlation is observed between the number of airborne spores of *C. cassiicola* and the diseased leaf area [25]. In addition, fungal spore size has been found to be an important virulence determinant in fungal pathogens including ascomycetes (*Beauveria bassiana*), basidiomycetes (*Cryptococcus neoformans*) or mucorales (*Mucor circinelloides*) [26,27,28]. However, the effect of *C*. *cassiicola* spore size on virulence against cucumber is still unknown.

Environmental factors, in particular temperature and moisture, are vital for phytopathogen sporulation. Some experiments have been conducted to examine the effects of temperature and moisture on the sporulation of fungal pathogens [29,30], and forecasting models have been developed based on the Richards model or Weibull model of temperature and the duration of high relative humidity for sporulation and infection [31,32,33]. The optimal ecological conditions for sporulation differ for each fungus. Lower temperatures are optimal for sporulation of *Pseudoperonospora cubensis*, *Phytophthora infestans*, and *B. cinerea*, corresponding to 16.2, 18, and 15–20 °C, respectively [33,34,35]. However, the optimal temperatures for sporulation of *Fusarium solani* and *Trichothecium roseum* are relatively high, at 25 and 28 °C, respectively [36,37]. Generally, plant pathogens sporulate over a wide range of air humidity. Furthermore, various species of powdery mildew may reach their highest sporulation under different RH. For example, the optimum moisture for *Erysiphe graminis* sporulation is 100% RH [38], whereas sporulation of *Sphaerotheca fuliginea* on squash favors 45–50% RH [39]. In addition, the moisture required for sporulation of *T. roseum* ranges from 70% to 95% RH [37], and the maximum sporulation of *F. solani* and *P. cubensis* is found at 100% RH [33,36]. Serious infection of *C. cassiicola* occurs under humid weather conditions and temperatures ranging from 20 to 30 °C throughout the day [5]. The optimum sporulation temperature for *C. cassiicola* isolated from tobacco is reported to be 27.5 to 30 °C [40]. However, recent studies have found that *C. cassiicola* isolated from cotton has the highest sporulation when cultured at ambient temperature or a constant 23 °C [41]. Moreover, the suitable temperature for sporulation of *C. cassiicola* isolated from noble sanchezia ranges from 20 to 28 °C [42]. These differences suggest that the optimal sporulation temperature of *C. cassiicola* is different for each host. Although a few reports have investigated the effect of temperature on *C. cassiicola* sporulation, until now, no detailed RH requirements for sporulation of *C. cassiicola* have been described.

Most previous experiments on sporulation have been performed in vitro or limited to field observations under uncontrolled conditions. Sporulation in vitro is a relatively simple system by which a fungus reproduces on a defined medium, which could provide insight into physiological, biochemical, and genetic phenomena that are difficult to study in complicated host−pathogen systems. However, in vitro studies of the sporulation patterns of fungi are often irrelevant to the results of in vivo studies [43]. Theoretical and practical studies of sporulation in vivo have been neglected for years, which is probably due to the misconception that sporulation in Petri dishes reflects the situation in nature. Thus, sporulation of plant pathogenic fungi in vivo urgently needs to be quantitatively evaluated.

An understanding of the conditions required for sporulation is therefore necessary to predict where secondary cycling of phytopathogenic fungi might occur and thus may provide insights into how fungal epidemics are initiated and maintained. Given that lower amounts of inoculum could retard both the rate and amount of disease increase, it is important to properly understand the conditions required for sporulation of *C. cassiicola* isolated from cucumber. The present study aimed to (i) study the dynamics of *C. cassiicola* sporulation on PDA and cucumber seedlings, (ii) produce quantitative data on how the sporulation of *C. cassiicola* is affected by different temperature and moisture regimes in vitro and in vivo, and (iii) determine the role of spore size of *C. cassiicola* in the virulence of cucumber. The experimental data are crucial to better understand the biology and epidemiology of *C. cassiicola*, screen cucumber germplasm for resistance, and develop effective disease management strategies.

## 2. Materials and Methods

### 2.1. Fungal Strain and Culture Media

*C. cassiicola* was collected from Langfang City, China, maintained in 40% glycerol stocks at −80 °C, and recultured on potato dextrose agar (PDA; potato 200 g/L, glucose 20 g/L, and agar 20 g/L) plates. Cucumber−leaf extract agar (CEA) was prepared using extracts of fresh cucumber leaves (200 g/L) following the procedure of Uppala et al. [44]. All media were autoclaved for 20 min at 121 °C, cooled to 50 °C in a water bath, and dispensed into sterile 85 mm Petri plates at 15 mL per plate.

### 2.2. Seedling Cultivation

A native cucumber (*Cucumis sativus* L.) cultivar ‘Zhongnong No. 6’ (China Vegetable Seed Technology Co., Ltd., Beijing, China) was used in this experiment. Seeds were soaked in distilled water in a conical flask for 30 min at 55 °C and germinated in the dark at 22 °C for 48 h. The germinated seeds were planted in 8 cm diameter plastic pots filled with sterile substrate. The pots were then kept in a greenhouse at 18–20 °C (night)/26–30 °C (day) under natural daylight conditions. Cucumber seedlings at the two true leaf stage were used for inoculation.

### 2.3. Inoculum Preparation

The inocula were prepared by incubating the fungal strain on PDA plates at 28 °C in the dark for 10 days. A spore suspension was collected from the cultures by adding 10 mL of sterilized water with 0.05% Tween 20 to each plate and gently scratching with a sterilized soft brush. These spores were filtered through four layers of sterile gauze into 50 mL conical tubes. The spore concentration was adjusted to 1 × 10^5^ spores/mL using a hemocytometer before inoculation.

### 2.4. Temperature and Moisture Conditions

The effects of temperature and moisture on the sporulation of *C. cassiicola* were investigated on PDA, CEA, and detached leaves in vitro and cucumber seedlings in vivo. For each in vitro and in vivo test, the sporulation was assessed at six temperatures (10, 15, 20, 25, 30, and 35 °C) with three moisture levels (75%, 85%, and 100% RH) per temperature, and a total of 18 temperature−moisture combinations were tested.

Temperatures were controlled using artificial climate incubators (RLD−260D−3; Ningbo le electrical instrument manufacturing Co. Ltd., Ningbo, China) both in vitro and in vivo, and the temperature control ranged from 0 °C to 50 ± 1 °C. In vivo test, moistures were also controlled by the incubators, and the controlled RH range was between 50 and 95 ± 5%. Different levels of moisture in the in vitro tests were achieved by using stable saturated solutions of reagent−grade salts (Shanghai Aladdin Bio−Chem Technology, Shanghai, China), which maintained specific vapor pressures at a given temperature in enclosed environments [45]. Briefly, in vitro moist chambers consisted of sterilized plastic boxes with a size of 350 × 135 × 85 mm (length × width × height) that were sealed with parafilm containing 300 mL of saturated salt solution to maintain a range of moisture values at each temperature; NaCl was used for 75% RH, KCl was used for 85% RH [46], and sterilized water was used for 100% RH. All temperature and moisture values were recorded by a portable electronic sensor (DL−WS20, Testing Technology Ltd., Hangzhou, China) and were adjusted to the required conditions 12 h before each experiment.

### 2.5. Sporulation Dynamics of C. cassiicola In Vitro and In Vivo

In vitro assay: Sporulation dynamics of *C. cassiicola* in vitro were examined on 85 mm diameter PDA Petri plates. Each plate was inoculated in the center with a 5 mm agar disc, topside down, from a five−day−old PDA culture and incubated at 28 °C in the dark. Radial growth and spore production were examined at 3, 6, 9, 12, 15, 18, and 21 days after incubation (DAI). For radial growth measurements, two perpendicular straight lines (diameters) were drawn on the bottom of each Petri plate. The crossing point coincided with the center of the initial 5 mm fungal disc. Colony diameters were measured on the perpendicular lines, and the mean colony diameter was obtained by averaging both transects. Net radial growth was obtained by subtracting 5 mm from the mean colony diameter. Spore productions were obtained using a whole mycelium harvest technique. For each Petri plate, the whole culture of *C. cassiicola* was scraped with a sterilized soft brush by adding 10 mL of sterilized water containing 0.05% Tween 20 and then filtered through four layers of cheesecloth to remove mycelial fragments. After vortexing for 5 s, the number of spores per mL of the filtrate was enumerated by using a hemocytometer under a ×100 microscope (NIKON 80i, Japan). Spore production numbers were expressed as spores per sq cm of mycelium. Each time point was replicated three times.

In vivo assay: Sporulation dynamics of *C. cassiicola* in vivo were examined on cucumber seedlings with TLS disease. Cucumber seedlings with two true leaves were six−point inoculated using 10 μL of spore suspension (1 × 10^5^ spores/mL) on the adaxial side of each true leaf with a micropipette. The inoculated seedlings were kept under 98% RH and 25 °C for 48 h and then incubated in the greenhouse at 15–18 °C (night)/24–27 °C (day) under natural daylight conditions. Five days later, seedlings exhibiting TLS disease symptoms were first blown by a small fan (20 cm in diameter) from different directions to remove any spores present on the leaf surface and then moved into in vivo moist chambers containing 500 mL distilled water and immediately sealed by Parafilm, then placed in artificial climate incubators (RLD−260D−3) maintained at 28 °C. The in vivo moist chamber, made of organic glass with a size of 450 × 350 × 250 mm (length × width × height), was prefilled with 500 mL distilled water and immediately sealed by Parafilm to maintain a high humidity of 100% RH. Spore production was examined at 0, 2, 4, 6, 8, 10, 12, 14, and 16 h after incubation (HAI). Briefly, spores were collected by placing Scotch tape on the surface of each true leaf (upper and lower sides). Twelve visual fields with dense aggregates of spores were selected for each replication, and the number of spores in the visual field was counted under a ×200 microscope. Observations and photographs were taken in glycerin−lactic acid (Biotopped, Beijing, China) with 0.15% acid fuchsin. Each time point was replicated three times.

### 2.6. Effects of Temperature and Moisture on Sporulation of C. cassiicola

PDA and CEA assays: The effects of temperature and moisture on the sporulation of *C. cassiicola* were determined on 85 mm diameter PDA and CEA Petri plates. Each PDA and CEA plate was prepared and inoculated in the center with a 5 mm agar disc, placed topside down, from a five−day−old PDA culture. Uncovered inoculated plates were placed in a moist chamber, which was supported 30 mm directly above the solution by a 300 × 120 mm stainless steel wire mesh, and all chambers were sealed by Parafilm immediately. These inoculated plates were then incubated at 18 temperature–moisture combinations in darkness. Radial growth, spore production, and spore size were examined at 12 DAI. Radial growth and spore production were measured as described above. Spore size was measured by Nano Measurer software (Version 1.2, Shanghai, China). The length and width of spores were examined by microscopy under 200× magnification. Each temperature and moisture combination was replicated three times.

Detached leaf assay: Healthy cucumber seedlings were inoculated at six points with a *C. cassiicola* spore suspension as described above. Diseased leaves were first blown by a small fan to remove spores on the leaf surface and then taken from uniformly infected seedlings using a sterile surgical knife at five DAI when typical symptoms of TLS disease were observed. To prevent leaf dehydration, the petioles were immediately wrapped with moistened absorbent cotton. These symptomatic leaves were then incubated at different temperature and moisture conditions in darkness. The leaves in moist chambers were placed 30 mm above the saturated salt solution as described previously. The number and size of spores on target spots of detached leaves were counted and measured at 12 HAI. Each temperature and moisture combination was replicated three times.

In vivo test: Cucumber seedlings infected with TLS disease without any spores present were prepared as described above. The seedlings showing typical TLS symptoms were sufficiently watered and then incubated in different temperature and moisture conditions in darkness. The first group was placed into an in vivo moist chamber that contained 500 mL of distilled water and sealed with Parafilm to maintain 100% RH. The other two groups were put into Perspex containers without distilled water but covered with a layer of dry gauze to avoid the effect of air movement in the incubators, and 75% and 85% RH were directly maintained by the artificial climate incubators. The Perspex containers were moved into the incubators randomly and assigned to one of the preset tested temperatures. The spore production and size were measured at 12 HAI as previously described. Each temperature and moisture combination was replicated three times.

### 2.7. Effects of Spore Size in the Virulence of C. cassiicola on Cucumber

The virulence of *C. cassiicola* spores with different size was assessed on cucumber seedlings. Firstly, cucumber seedlings were inoculated with *C. cassiicola* spore suspension (1 × 10^5^ spores/mL) obtained from PDA plates and placed in a greenhouse at 25 °C under 98% RH and for 5 d. Subsequently, seedlings exhibiting TLS disease symptoms were blown by a small fan to remove any spores present and immediately moved into incubators maintained at 100%, 85%, and 75% RH at 28 °C for 12 h. The newly produced spores on the cucumber leaves were examined under a ×200 microscope, washed with distilled water, and removed with a soft brush to make spore suspensions. A hemocytometer was used to adjust the concentration of the suspensions to 1 × 10^3^ spores/mL. Thirdly, healthy cucumber seedlings were four−point inoculated using the resulting suspensions. Sterilized distilled water was used for negative control inoculations. Inoculated seedlings were maintained in a greenhouse at 25 °C with 98% RH for 48 h. After that, the inoculated seedlings were moved out and incubated in a greenhouse maintained at 15–18 °C (night)/24–27 °C (day) under natural daylight conditions and monitored daily for the presence or absence of symptoms. Lesion expansion was assessed at 7 DAI. Lesion dimensions were measured in two perpendicular directions and used to determine the lesion area. Each size of spores was replicated three times.

### 2.8. Data Analysis

Data on the net radial growth and spore production and size of *C. cassiicola* were recorded as the mean ± standard deviation (SD) of three independent biological replicates. Statistical analysis was conducted by either one− or two−way analysis of variance (ANOVA) using generalized linear models (GLMs) for the effects of different incubation times, temperatures and moisture levels on *C. cassiicola* sporulation (IBM SPSS, version 24, Chicago, IL, USA). Mean separations were performed using Tukey’s test at *p* = 0.05 and indicated with alphabetical notation.

## 3. Results

### 3.1. Sporulation Dynamics of C. cassiicola In Vitro

**Figure 1:**
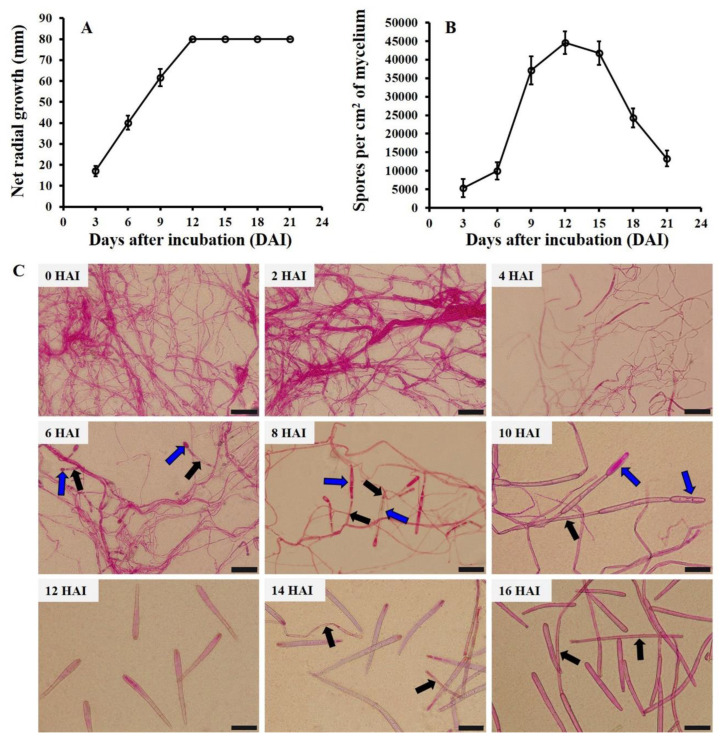
Radial growth (**A**) and sporulation production (**B**) of *Corynespora cassiicola* incubated on potato dextrose agar (PDA) at 28 °C in darkness and examined at 3, 6, 9, 12, 15, 18 and 21 days after incubation (DAI). Radial growth was measured on two perpendicular axes for each plate. The spores were counted using a whole mycelium harvest technique by a hemocytometer. Data points are the means of three replicates, and error bars represent standard deviations. (**C**) Sporulation dynamics of *C*. *cassiicola* on cucumber seedlings incubated at 28 °C and 100% RH in darkness and examined at 0, 2, 4, 6, 8, 10, 12, 14, and 16 h after incubation (HAI). Spores were collected by placing Scotch tape on the surfaces of cucumber leaves (upper and lower sides). Blue arrowheads indicate the spores remain on the conidiophores. Black arrowheads indicated the conidiophores (Scale bars, 50 μm).

*C. cassiicola* was inoculated on PDA at 28 °C in the dark. Radial growth and spore production on PDA were examined at 3, 6, 9, 12, 15, 18, and 21 DAI. Generally, net radial growth increased at first and then remained constant. The net colony diameter reached a maximum of 80 mm at 12 DAI, which remained constant until 21 DAI (Figure 1A). Concomitantly, the spore production number first increased and then decreased with the extension of incubation time. Low spore production of *C. cassiicola* was found at three DAI, with approximately 5.35 × 10^3^ spores per sq cm of mycelium. The highest spore production was detected at 12 DAI, with 4.45 × 10^4^ spores per sq cm of mycelium. After that, a strong reduction in spore production was observed from 15 DAI to 21 DAI, and the spore production decreased to 1.33 × 10^4^ spores per sq cm of mycelium at 21 DAI (Figure 1B). Thus, 12 DAI would be the most suitable time to investigate the effects of different temperature and moisture conditions on the sporulation of *C. cassiicola* in vitro.

### 3.2. Sporulation Dynamics of C. cassiicola In Vivo

Cucumber seedlings were inoculated at six points with a *C. cassiicola* suspension on the adaxial side of leaves and incubated at 28 °C and 100% RH in darkness. Spore production on diseased spots was examined at 0, 2, 4, 6, 8, 10, 12, 14, and 16 HAI. Mycelia of *C. cassiicola* began to specialize into conidiophores at four HAI. Conidiophores bearing small immature spores were observed at six HAI. Mature spores, which fell off from the conidiophores, were found at 12 HAI (Figure 1C), with a mean of 108.47 spores per visual field. After that, there was a rapid increase in spore number from 12 HAI to 16 HAI, which reached 319.75 spores per visual field at 16 HAI. Consequently, 12 HAI was chosen as the optimal time to investigate the effects of different levels of temperature and moisture on the sporulation of *C. cassiicola* in vivo.

### 3.3. Effects of Temperature and Moisture on Spore Production

Two−way ANOVA revealed that temperature, moisture, and their interaction significantly influenced the spore production of *C. cassiicola* in vitro and in vivo (*p* < 0.05). On PDA and CEA, temperature was the most important factor, explaining 81.62% and 66.48% of the total variance, followed by moisture and the interaction between these two parameters, explaining 9.15% and 8.84% of the total variance on PDA and 20.10% and 12.18% of the total variance on CEA, respectively. On detached leaves and cucumber seedlings, spore production was strongly influenced by both temperature and moisture, corresponding to 41.08% and 36.78% of the total variance on detached leaves and 36.78% and 43.65% of the total variance on cucumber seedlings, respectively (Table S1).

**Figure 2:**
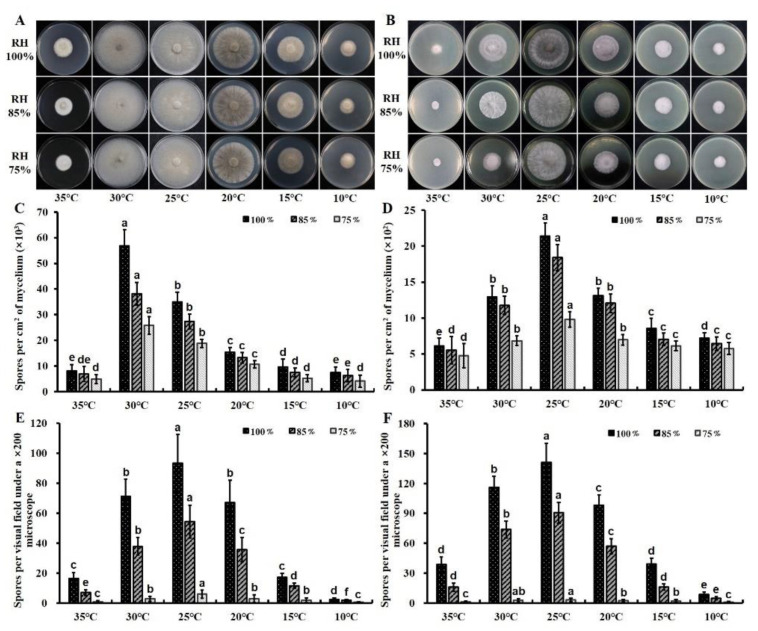
Colony morphology and sporulation production of *Corynespora cassiicola* on different substrates at six temperatures (10, 15, 20, 25, 30, and 35 °C) with three moisture levels (75%, 85%, and 100% RH) per temperature. (**A**,**B**) Colonies of *C*. *cassiicola* on potato dextrose agar (PDA) and cucumber−leaf extract agar (CEA) at 12 days after incubation (DAI). (**C**,**D**) Sporulation production of *C*. *cassiicola* on PDA and CEA at 12 DAI. Spore production number was reported as spores per sq cm of mycelium. (**E**,**F**) Sporulation production of *C*. *cassiicola* on detached and living leaves of cucumber at 12 h after incubation. Spore production number was reported as spores per visual field under a ×200 microscope. Data are means of three experimental repetitions. Different letters represent significant (*p* < 0.05) differences with post hoc multiple comparisons according to Tukey’s test.

With respect to temperature, incubation at 30 °C resulted in the maximum spore production, corresponding to 4.03 × 10^4^ spores per sq cm of mycelium on PDA, which was significantly higher (*p* < 0.05) than that under the other five temperatures. Fewer spores were observed with the stepwise increase or decrease of 5 °C. Spore productions at extremely marginal temperatures of 10 °C and 35 °C were fairly low, with approximately 5.97 × 10^3^ and 6.62 × 10^3^ spores per sq cm of mycelium, respectively (Figure 2A,C). On CEA, detached leaves, and cucumber seedlings, the highest spore productions were found at 25 °C, corresponding to 1.65 × 10^3^ spores per sq cm of mycelium on CEA and 51.25 and 78.38 spores per visual field on detached leaves and cucumber seedlings, respectively. Fewer spores were obtained with a stepwise change of 5 °C. Significantly lower spore productions were observed at 10 °C and 35 °C on CEA, which were 6.48 × 10^2^ and 5.48 × 10^2^ spores per sq cm of mycelium, respectively (Figure 2B,D). Relatively lower spore amounts were produced at 10 °C and 35 °C on detached leaves and cucumber seedlings, respectively, with approximately 1.53–18.69 spores per visual field (Figure 2E,F).

In relation to moisture, lower spore productions of *C. cassiicola* were observed with a reduction from 100% to 75% RH in vitro and in vivo. The largest spore production was detected at 100% RH, with averages of 2.21 × 10^4^ spores per sq cm of mycelium on PDA, 1.16 × 10^3^ spores per sq cm of mycelium on CEA, 44.66 spores per visual field on detached leaves, and 73.69 spores per visual field on cucumber seedlings. As the moisture decreased to 75% RH, the spore productions were 1.16 × 10^4^ and 6.72 × 10^2^ spores per sq cm of mycelium on PDA and CEA, respectively (Figure 2C,D). Few spores were formed on detached leaves and cucumber seedlings at 75% RH, with approximately 2.46 and 2.15 spores per visual field, respectively (Figure 2E,F).

### 3.4. Effects of Temperature and Moisture on Spore Size

**Figure 3:**
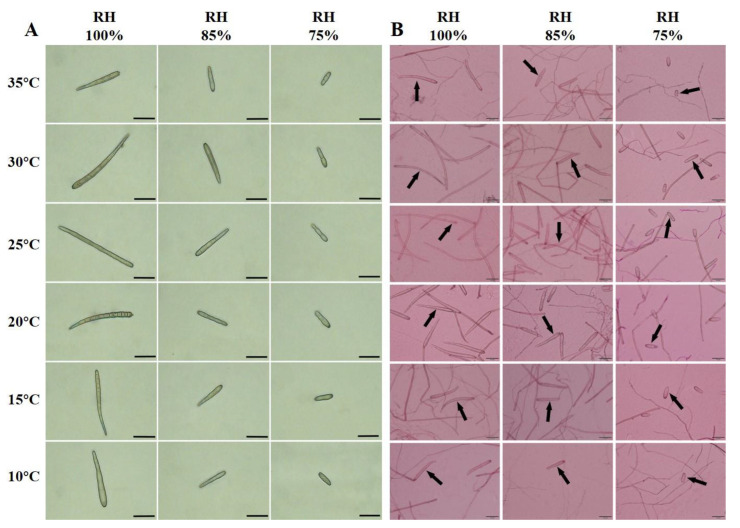
Morphological characteristics of *Corynespora cassiicola* on cucumber−leaf extract agar (CEA) (**A**) at 12 days after incubation (DAI) and cucumber seedlings (**B**) at 12 h after incubation (HAI), which were assessed at six temperatures (10, 15, 20, 25, 30, and 35 °C) with three moisture levels (75%, 85%, and 100% RH) per temperature. Black arrowheads represented the spores (Scale bars, 50 μm).

The length and width of *C. cassiicola* spores on CEA, detached leaves, and cucumber seedlings were significantly affected by temperature and moisture (*p* < 0.05) (Figure 3 and Figure S1B), while no significant differences in spore size were observed among different temperature and moisture conditions in the PDA assay (*p* > 0.05) (Figure S1A).

**Figure 4:**
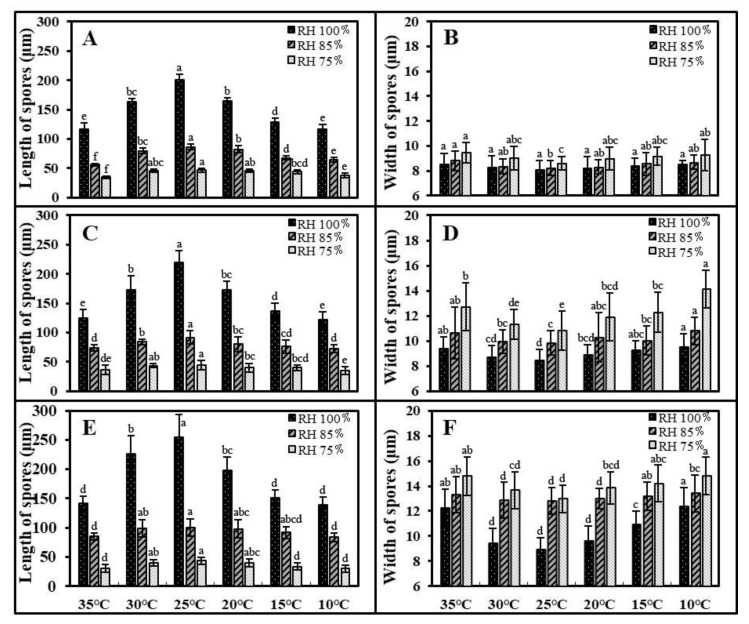
Spore size of *Corynespora cassiicola* affected by different temperatures and moisture levels on CEA (**A**,**B**), detached leaves (**C**,**D**) and cucumber seedlings (**E**,**F**). Data are means of three experimental repetitions. Bars with different letters are significantly different (*p* < 0.05) according to multiple comparisons with Tukey’s test.

Moisture was the most important factor affecting spore size, which accounted for 84.52%, 84.86%, and 83.09% of the total variance in length and 44.72%, 73.10%, and 66.67% of the total variance in width on CEA, detached leaves and cucumber seedlings, respectively (Table S1). Generally, as moisture decreased from 100% to 75% RH, the length of spores decreased gradually, while the width of spores increased substantially. Specifically, the longest−narrowest spores were formed at 100% RH, with averages of 148.53 × 8.35, 158.01 × 9.05, and 184.88 × 10.59 μm on CEA, detached leaves and cucumber seedlings, respectively. The shortest−widest spores were formed at 75% RH, with averages of 42.56 × 9.09, 39.94 × 12.19, 36.18 × 14.04 μm on CEA, detached leaves, and cucumber seedlings, respectively (Figure 3 and Figure 4).

Regarding the temperature, the length of spores was significantly influenced by temperature (*p* < 0.05), and significant differences were observed in the width of spores on detached leaves and cucumber seedlings (*p* < 0.05) but not on CEA (*p* > 0.05). Overall, the longest spores were formed at 25 °C, corresponding to 111.47 μm, 118.70 μm, and 132.73 μm on CEA, detached leaves, and cucumber seedlings, respectively. A significant decrease in spore length was observed with a stepwise change of 5 °C, similar to spore yield. The shortest spores were formed at 35 °C or 10 °C, which were 69.05 μm, 77.37 μm, and 84.19 μm on CEA, detached leaves, and cucumber seedlings, respectively. Concomitantly, the narrowest spores were produced at 25 °C, while the widest spores were produced at 35 °C or 10 °C (Figure 3 and Figure 4).

### 3.5. The Role of Spore Size in the Virulence of C. cassiicola on Cucumber

**Figure 5:**
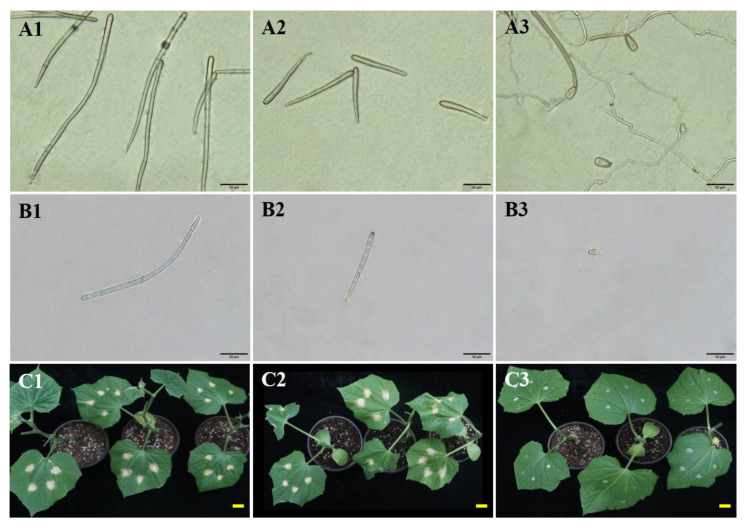
Influence of spore size on the virulence of *Corynespora cassiicola* in cucumber. (**A1**–**A3**) Morphological characteristics of *C. cassiicola* produced on the cucumber leaves incubated under 100%, 85%, and 75% RH at 28 °C for 12 h; (**B1**–**B3**) Spore suspensions of *C*. *cassiicola* collected from the cucumber leaves incubated under 100%, 85%, and 75% RH at 28 °C for 12 h; (**C1**–**C3**) At 7 days after incubation (DAI), symptoms of cucumber seedlings caused by spore suspensions collected from the cucumber leaves incubated under 100%, 85%, and 75% RH (Scale bars, 10 mm).

The spores of three different sizes (large, 282.20 μm; medium, 115.31 μm; small, 38.47 μm) of *C. cassiicola* produced under 100%, 85%, and 75% RH at 28 °C (Figure 5A1–A3,B1–B3) were evaluated on their virulence to cause TLS disease on cucumber. Spore size had a significant effect on the virulence of *C*. *cassiicola* in cucumber (*p* < 0.05). The large and medium spores germinate, whereas the small spores do not (Figure S2). Leaf spot symptoms caused by the large and medium spores, respectively, obtained under 100% and 85% RH were observed on the cucumber seedling at three DAI (Figure 5C1,C2). The lesion area caused by the large and medium spores was not significantly different (*p* > 0.05), with averages of 23.44 and 22.89 mm^2^, respectively. Cucumber seedlings inoculated with the small spores obtained under 75% RH and the control seedlings inoculated with sterile water were asymptomatic (Figure 5C3).

## 4. Discussion

Spores are produced in large quantities by *C. cassiicola*, representing the main form of secondary spread in the field, as they are easily dispersed by airflows or water jets [23,25]. Several studies have been conducted in vitro to evaluate the effects of substrate, light, temperature, pH, nutritional conditions, and stress on the sporulation of *C*. *cassiicola* [41,47,48,49]. Nevertheless, none of these studies reported quantitative data on sporulation under different regimes of temperature and moisture. Moreover, the sporulation patterns resulting from in vitro assays are often irrelevant to those in vivo [43]. This is the first study focused on the sporulation of *C. cassiicola* from cucumber on both artificial and natural matrices in relation to temperature and moisture.

Temperature is found to be a crucial factor that influences the amount of spore reproduction. At 28 °C which is favored by *C*. *cassiicola* in cucumbers, the spore generation peak was at 12 DAI for plates, and mature spores fell off from the conidiophores at 12 HAI for seedlings. Since the less favorable temperature reduces the activity of physiological processes and contributes to a slower metabolism, it usually requires more time for spores to reach the generation peak on plates and mature on seedlings. In general, when the time for sample collection is less than 12 DAI and 12 HAI, the more suitable temperature for *C*. *cassiicola* development, the more spores will mature and be released in a shorter period of time. When the sampling time is more than 12 DAI, sporulation efficiency at 30 °C on PDA will be restricted by the area of the Petri dishes and the insufficient amount of nutrients due that the mycelia reached the edge of the Petri dishes (see Figure 2A). The present experiment revealed that *C. cassiicola* produced spores at temperatures ranging from 10 to 35 °C. The pathogen produced significantly larger amounts of spores at temperatures from 20 to 30 °C than at other temperatures, and the largest spore production was inspected on cucumber TLSs incubated at 25 °C. The temperature range is also confirmed by earlier research [50]. However, the optimum sporulation temperature of *C. cassiicola* isolated from cucumber is lower than that reported on rubber trees [51] and higher than that reported on cotton [41], which is likely due to the difference in host specificity and geographic distribution. In the present study, the effects of temperature on sporulation were more significant in vitro than in vivo, which is probably because the moisture content is very high in the Petri dishes at the initial stage of incubation [52]. According to the theory of compensation, free medium water may weaken the effect of air humidity on sporulation [43]. In addition, sporulation on artificial substrates could be restricted by variation in the nutrient content with long−term exposure to a low RH [53].

The optimum sporulation temperature for *C. cassiicola* was 30 °C on PDA and 25 °C on CEA, detached cucumber leaves, and living plants, which indicates that sporulation is also significantly influenced by the incubation substrate. Greater spore productions were detected on the artificial PDA (1.67 × 10^4^ spores per sq cm of mycelium) than on the natural CEA (9.50 × 10^2^ spores per sq cm of mycelium). The result is similar to those of previous studies; the maximum sporulation of *C. cassiicola* from flue−cured tobacco is also observed on PDA [40], and for *Aspergillus flavus*, the mean numbers of spores are higher on artificial substrates than on natural substrates [54]. However, in other studies on *Cercospora janseana*, the plant−based medium stimulates sporulation [44]. These differences might be due to the different nutritional mechanisms of these pathogens. *C. cassiicola* and *A. flavus* can be saprotrophic, hemibiotrophic, or biotrophic, whereas *C. janseana* is generally an obligate parasite that grows very slowly and does not sporulate well on PDA [55]. In the present study, it is worth noting that the optimum temperature for sporulation on PDA was higher than that on the other three substrates. While a previous study showed that *C. cassiicola* is primarily found in the tropics and subtropics [1], there is a real possibility that diseases caused by *C. cassiicola* will become increasingly serious in subtropical and temperate areas as global warming worsens.

Moisture is a more important factor than temperature in influencing the sporulation of *C. cassiicola* on living cucumber plants. In the current research, higher moisture induced more sporulation. The maximum spore production occurred in the environment with saturated humidity (100% RH), and only a few spores were formed at 75% RH. This coincides well with the environmental conditions favorable for TLS disease outbreaks in the field. A similar pattern was found in which sporulation is highly dependent on climatic conditions, in particular the availability of high environmental moisture [33,36,37]. Furthermore, spores of *C. cassiicola* were observed after 6 h of incubation at 100% RH and 28 °C in darkness. The number of spores produced by the pathogen on cucumber chlorotic lesions increased exponentially with the extension of the incubation period. Several Oomycetes, such as *Pseudoperonospora cubensis*, *Peronospora destructor*, and *Phytophthora colocasiae*, produce sporangia within a wetting period as short as 2 to 4 h [33,56,57]. However, the wetting periods required for sporulation of most parasitic fungi are much longer [58,59]. The number of spores produced on PDA first increased and then decreased with the extension of incubation time. This could be explained by space limitations, exhaustion of nutritional substances, insufficient aeration, and autolysis of the pathogenic mycelium due to long−term incubation [43,60,61].

Spore size is significantly influenced by moisture. Longer spores were always produced in the environment with 100% RH rather than at the other two moisture conditions on CEA, detached cucumber leaves, and living cucumber leaves. The spores produced on PDA were generally smaller than those observed on host lesions and did not differ significantly with the different humidity levels. This could be because some nutrients extracted from cucumber or in cucumber itself are favored for sporulation of *C. cassiicola*. Several studies have reported that spore dimensions are affected by the type of culture medium, host species, and resource constraints [62,63]. Our work showed that larger spores of *C*. *cassiicola* displayed significantly enhanced virulence against cucumber, which is confirmed by previous studies in fungal pathogens [26,27,28]. Li et al. [64] stated that larger spores lead to a faster germination rate that, in turn, correlates with a more virulent phenotype, which associates with the results of this study. Furthermore, spore size plays a significant role in taxonomy, facilitating the identification of species.

Mature spores of *C. cassiicola* fell off from the conidiophores after incubation for 12 h at 100% RH and 28 °C in darkness. In the fields, such long periods of high humidity do not usually occur, except during cloudy, rainy, and snowy weather. Some pathogens overcome the lack of prolonged humid conditions by using several short wet periods (usually at night) interrupted by dry intervals during the day, which are known as interrupted wetting period regimes [18,59]. Conidiophores with or without immature spores formed in one night could withstand unfavorable conditions the following day and continue to develop during the subsequent night or the reappearance of humid conditions [65].

## 5. Conclusions

This study demonstrates that temperature and moisture affect sporulation of *C*. *cassiicola* in vitro and in vivo, and shows that spore size is an important virulence determinant in the fungus. The suitable temperature and moisture for the fungus sporulation is 25–30 °C and 100% RH because they allow higher spore production, larger spore size, and greater spore virulence. Our work is crucial to predict how environmental factors such as temperature and moisture can increase the risk of disease outbreaks in the field. Moreover, the quantitative data obtained in this study represent pillars of knowledge for farmers to optimize agricultural practices in cucumber growing areas. Further studies in our laboratory are underway to determine the molecular mechanism of spore size related to the virulence in *C*. *cassiicola*. In addition, knowledge of the regulatory network could lay a foundation for the disease−resistant breeding of cucumber.

## Data Availability

Not applicable.

## Supplementary Materials

The following supporting information can be downloaded at: https://www.mdpi.com/article/10.3390/biology11111675/s1, Table S1: Analysis of variance considering the effects of temperature, moisture and their interaction on sporulation of the tested strain in this study; Figure S1: Morphological characteristics of *Corynespora cassiicola* on potato dextrose agar and cucumber detached leaves; Figure S2: Germination of different size spores.
