# Supplementary material for: Temperature and Humidity Regulate Sporulation of Corynespora cassiicola That Is Associated with Pathogenicity in Cucumber (Cucumis sativus L.)
Source: Biology (Basel). 2022 Nov 18;11(11):1675. doi: 10.3390/biology11111675 (PMC9687187; doi:10.3390/biology11111675)
Supplement: Supplementary file 1 [file biology-11-01675-s001.zip › biology-1935521-Supplementary.pdf]

Table S1. Analysis of variance considering the effects of temperature, moisture and their interaction on sporulation of *Corynespora cassiicola*.

Sporulation	Substrate	Percent variance explained		
		Temperature	Moisture	Interaction
Spore production	PDA	81.62%	9.15%	8.84%
	CEA	66.48%	20.10%	12.18%
	detached leaves	41.08%	36.78%	21.77%
	cucumber seedlings	36.78%	43.65%	19.48%
Spore length	PDA	N.S.	N.S.	N.S.
	CEA	9.56%	84.52%	5.70%
	detached leaves	7.62%	84.86%	6.96%
	cucumber seedlings	7.62%	83.09%	7.41%
Spore width	PDA	N.S.	N.S.	N.S.
	CEA	19.35%	44.72%	1.54%
	detached leaves	14.88%	73.10%	5.57%
	cucumber seedlings	17.13%	66.67%	7.18%

This table shows how much temperature, moisture and their interaction explain the total variance in spore production, length and width of *C. cassiicola* in vitro and in vivo.
PDA, potato dextrose agar; CEA, cucumber-leaf extract agar.
The percentage figures are calculated from the type III sum of squares in ANOVA table gotten through SPSS (version 24).
N.S. (not significant) means that no significant differences were observed among different levels of temperature and moisture.

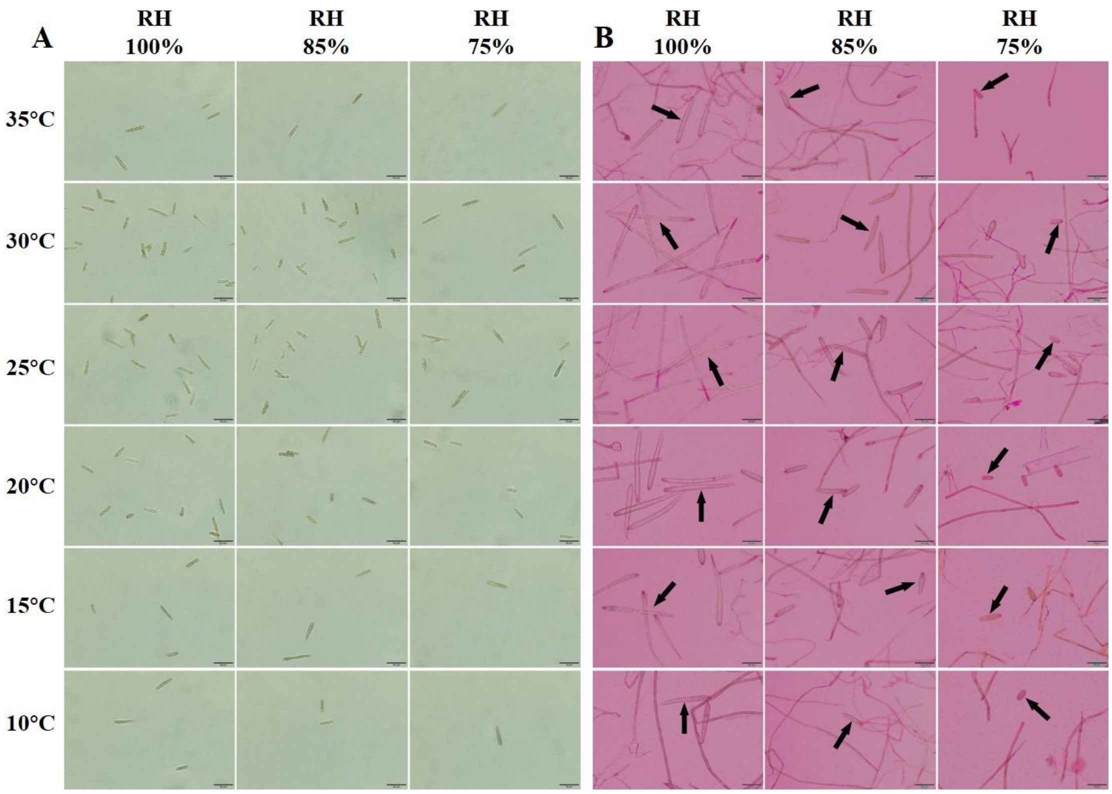

Figure S1. Morphological characteristics of *Corynespora cassiicola* on potato dextrose agar (PDA) (**A**) at 12 days after incubation (DAI) and cucumber detached leaves (**B**) at 12 hours after incubation (HAI), which were assessed at six temperatures (10, 15, 20, 25, 30, and 35 °C) with three moisture levels (75%, 85%, and 100% RH) per temperature. Black arrowheads represented the spores (Scale bars, 50 μm).

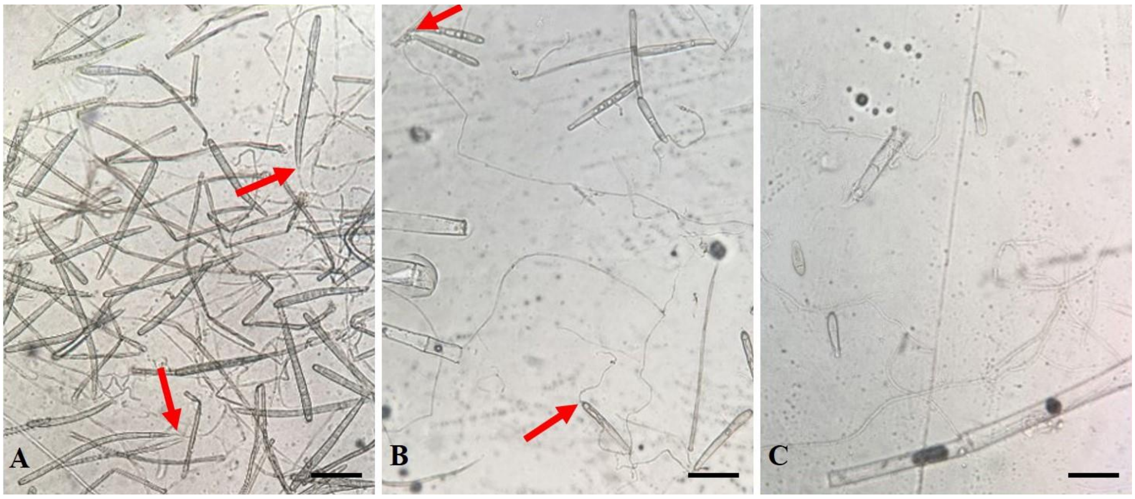

Figure S2. Germination of different size spores. (**A**) Large spores germinated and produced invasive hyphae; (**B**) Medium spores germinated and produced invasive hyphae; (**C**) Small spores did not germinate. Red arrowheads indicated the ends at which spores germinated (Scale bars, 50 μm).
